# Effects of cadmium chloride on mouse inner medullary collecting duct cells

**DOI:** 10.2478/intox-2013-0025

**Published:** 2013-09

**Authors:** Eun-Kee Park, Sally K. Mak, Bruce D. Hammock

**Affiliations:** 1 Department of Medical Humanities and Social Medicine, College of Medicine, Kosin University, Busan, Republic of Korea; 2 Physiological Genomics Group, Department of Animal Science, University of California, Davis, CA, USA; 3 Department of Entomology and UCD Comprehensive Cancer Center, University of California, Davis, CA, USA

**Keywords:** cadmium chloride, cytotoxicity, kidney, mIMCD3 cells

## Abstract

Cadmium is a known renal toxin. The cytotoxic effect of cadmium chloride (CdCl_2_) was evaluated on renal inner medullary collecting duct cells (mIMCD3). The 24 hr LC_50_ value for CdCl_2_ in mIMCD3 cells was 40 µM. The present study showed that mIMCD3 cells were sensitive to CdCl_2_ exposure.

## Introduction

Cadmium exposure is a public health concern for renal diseases, even at low levels of exposure (Ferraro *et al*., 2010; Kobayashi *et al*., 2009; Thomas *et al*., 2009) because the kidney is the organ most sensitive to cadmium toxicity (Järup *et al*., 1998). Most renal cell studies have focused less on the inner medulla although it is often exposed to high concentrations of common nephrotoxins (Burg, 2002; Rocha *et al*., 2001; Yancey *et al*., 1982). Renal inner medullary collecting duct cells (mIMCD3), which are an immortalized cell line derived from the mouse renal inner medulla, have proven a useful system to investigate effects of nephrotoxins (Cai *et al*., 2003; Kojima *et al*., 2011; Park *et al*., 2007; Park *et al*., 2008; Schenk *et al*., 2010). The present study investigated the effect of cadmium chloride on mIMCD3 cells.

## Materials and methods

### Cell culture and chemicals

This experiment was performed as previously described (Park *et al*., 2007; Park *et al*., 2008). All reagents for cell culture were purchased from Life Technologies (Carlsbad, CA, USA). Briefly, mIMCD3 cells were grown in the presence of 45% Ham's F-12, 45% Dulbecco's modified Eagle's medium, 10% fetal bovine serum (FBS), 10 milliunits/ml penicillin and 10 µg/ml streptomycin. The final osmolality of isosmotic medium was 300±5 mosmol/kg medium, which was confirmed by a microosmometer (Model 3300, Advanced Instruments, Norwood, MA, USA). Cells were grown at 37 °C and 5% CO_2_. Cadmium chloride (CdCl_2_) was purchased from Sigma (St. Louis, MO, USA) and dissolved in Milli-Q water (Millipore, Bedford, MA, USA) freshly.

### Cytotoxicity assays

Cell viability to determine the cytotoxic effect of CdCl_2_ was carried out using the 3-(4,5-dimethylthiazol-2-yl)-2,5-diphenyl tetrazolium bromide (MTT) assay (Roche Applied Science, IN, USA) as described previously (Park *et al*., 2007; Park *et al*., 2008). Briefly, mIMCD3 cells were grown, trypsinized, and seeded evenly with 100 µL of medium into each well of a flat-bottomed 96-well cell culture plate (Nalge-Nunc, Rochester, NY, USA). Once confluent, the desired concentrations of CdCl_2_ for testing were diluted from a stock solution, added to the wells and incubated in a humidified incubator of 5% CO_2_ at 37 °C for 24 hr. Controls were the cells without CdCl_2_ treatment. MTT assay was performed according to the manufacture's instruction. Briefly, 10 µL MTT reagent was added into each well and cells incubated for 4 hr, followed by addition of 100 µL of solubilization solution into each well. After 24 hr incubation, the ratio of absorbance at 560 nm versus 750 nm was measured with a SpectraFluor Plus microplate reader (Tecan, Durham, NC, USA). This ratio represented a measure of viable cells in each well and this ratio was normalized to controls that were run in parallel in the 96-well plate. Each condition was repeated in 8 wells and experiments were independently replicated 5 times. The concentration at which after 24 hr half of the cells for each of concentration of the toxins tested were viable (LC_50_) was determined. The results were expressed as percentage of cell survival compared to the control. Data were presented as mean ± S.E.M.

## Results and discussion

**Figure 1:**
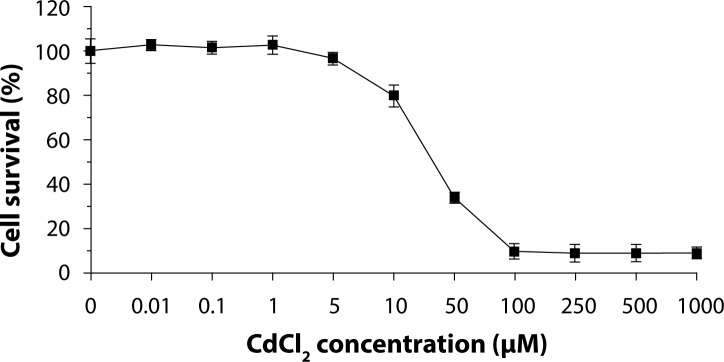
Cytotoxicity caused by CdCl_2_ in mIMCD3 cells in normal isosmotic (300 mosmol/kg) medium. Data are expressed as % cell survival compared to control (5 independent experiments).

Control (water) had no influence on the survival of mIMCD3 cells. The 24 hr LC_50_ value for CdCl_2_ in mIMCD3 cells was 40 µM in this experiment (Figure 1). The results of this study demonstrated that CdCl_2_ is directly toxic to mIMCD3 cells, which are well suited for this study. Previous studies reported that cadmium chloride (CdCl_2_) caused damage to the proximal tubular epithelium of the mammalian kidney (Järup, 2002; Prozialeck *et al*., 1993; Van Vleet & Schnellmann, 2003). A similar toxic effect of CdCl_2_ in LLC-PK1 cells (pig renal proximal tubule cell line) was found with a 24 hr LC_50_ value of 50 µM (Gennari *et al*., 2003). The cell viability at 9 hr was decreased by 38% and 45% at 25 and 50 µM CdCl_2_, respectively (Gena *et al*., 2010). CdCl_2_ was reported to cause DNA strand breaks, lipid peroxidation, reactive oxygen species, induction of necrosis and apoptosis, and to inhibit Na, K-ATPase (Kinne-Saffran *et al*., 1993; Mao *et al*., 2007; Mao *et al*., 2011; Valverde *et al*., 2001).

Overall, the present study revealed that cadmium chloride has a toxic effect on inner medulla areas and that mIMCD3 cells could be suited for studying the mechanisms related to CdCl_2_ toxicity.
